# Forty Years after the Discovery of Its Nucleolytic Activity: [Cu(phen)_2_]^2+^ Shows Unattended DNA Cleavage Activity upon Fluorination

**DOI:** 10.1002/chem.202004594

**Published:** 2021-01-22

**Authors:** Carsten Lüdtke, Sebastian Sobottka, Julian Heinrich, Phil Liebing, Stefanie Wedepohl, Biprajit Sarkar, Nora Kulak

**Affiliations:** ^1^ Institut für Chemie und Biochemie Freie Universität Berlin Fabeckstr. 34/36 14195 Berlin Germany; ^2^ Institut für Chemie Otto-von-Guericke-Universität Magdeburg Universitätsplatz 2 39106 Magdeburg Germany; ^3^ Institut für Chemie und Biochemie Freie Universität Berlin Arnimallee 22 14195 Berlin Germany; ^4^ Institut für Anorganische Chemie Universität Stuttgart Pfaffenwaldring 55 70569 Stuttgart Germany

**Keywords:** artificial nucleases, copper complexes, cytotoxicity, fluorination, phenanthroline

## Abstract

[Cu(**phen**)_2_]^2+^ (**phen**=1,10‐phenanthroline) is the first and still one of the most efficient artificial nucleases. In general, when the **phen** ligand is modified, the nucleolytic activity of its Cu^II^ complex is significantly reduced. This is most likely due to higher steric bulk of such ligands and thus lower affinity to DNA. Cu^II^ complexes with **phen** ligands having fluorinated substituents (F, CF_3_, SF_5_, SCF_3_) surprisingly showed excellent DNA cleavage activity—in contrast to the unsubstituted [Cu(**phen**)_2_]^2+^—in the absence of the otherwise required classical, bioabundant external reducing agents like thiols or ascorbate. This nucleolytic activity correlates well with the half‐wave potentials *E*_1/2_ of the complexes. Cancer cell studies show cytotoxic effects of all complexes with fluorinated ligands in the low μm range, whereas they were less toxic towards healthy cells (fibroblasts).

## Graphical abstract

**Activation by fluorination**: By introducing fluorine‐containing substituents in the 1,10‐phenanthroline ligands of a [Cu(phen)_2_]^2+^ complex, nucleolytic activity in the absence of classical, bioabundant external reducing agents is observed. Such an activity is unknown for the unsubstituted [Cu(phen)_2_]^2+^, the first and otherwise one of the most efficient artificial nucleases. Also, the cytotoxicity profiles of the new complexes were different—though less toxic, cancer cells were more harmed than healthy cells.

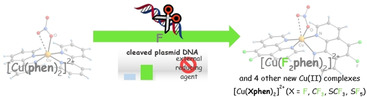

A few decades ago, Sigman et al. discovered the nucleolytic activity of bis(1,10‐phenanthroline)copper(II) [Cu(**phen**)_2_]^2+^ towards dsDNA in the presence of O_2_ and 3‐mercaptopropionic acid as a reducing agent. The active species was shown to be the corresponding Cu^I^ complex. Thus, reducing agents are mandatory for initiating the generation of reactive oxygen species (ROS), which are required for exerting oxidative damage to DNA.^[1]^ Different groups have tried to enhance and manipulate this nucleolytic activity, for example, via substitution of hydrogen atoms on the ligand scaffold. DNA cleavage can even be inhibited as by 2,9‐dimethylphenanthroline (neocuproine), which scavenges Cu^I^ and blocks the redox cycle involving the production of ROS. All [CuL_2_]^2+^ species (L=**phen** derivatives like dipyrido[3,2‐d:2’,3’‐f]quinoxaline (dpq) and dipyrido[3,2‐a:2’,3’‐c]phenazine (dppz)) investigated until today required external reducing agents for oxidative DNA cleavage like thiols or ascorbate.[^2^, ^3^, ^4^, ^5^] The only reported examples where no addition of external reductants was necessary were obtained by linkage of two [Cu(**phen**)_2_]^2+^ moieties by aliphatic and aromatic dicarboxylates. A “self‐activation” mechanism for ROS formation based on a ligand radical species was proposed.^[6]^

Our group has focused on the manipulation of the redox properties of [Cu(**phen**)_2_]^2+^ complexes via substitution of the ligand scaffold with fluorine or fluorine‐containing groups. In this report, we are presenting the synthesis of new homoleptic Cu^II^ complexes with **phen** ligands having fluorine or fluorinated substituents at positions 5 and/or 6 (Figure 1). The interaction of these complexes with DNA, their nucleolytic activity as well as their cytotoxic properties are described herein. Fluorine‐containing **phen** derivatives were obtained via Skraup synthesis as described previously, starting from prefunctionalized nitroaniline and nitroquinoline precursors.^[7]^ Such **phen** complexes are barely known to the literature even with other metals. To the best of our knowledge, the only examples up to date represent a homoleptic Cu^I^ complex with two to four CF_3_ groups in the **phen** moiety and Pd^II^ complexes [PdCl_2_(L)] and [PdCl_2_(PPh_3_)(L)] with L=4,7‐dichloro‐5‐fluoro‐2,9‐dimethyl‐1,10‐phenanthroline.[^8^, ^9^] Besides that, theoretical investigations have been carried out for Ru complexes with 5,6‐difluoro‐1,10‐phenanthroline (**F_2_phen**) and 5‐trifluoromethyl‐1,10‐phenanthroline (**CF_3_phen**).[^10^, ^11^]

**Figure 1:**
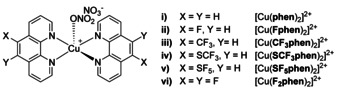
Cu^II^ complexes with fluorinated **phen** ligands. For i)–v) only one of two possible isomers generated by asymmetric substitution at positions 5 and 6 of the **phen** moiety is shown.

The corresponding Cu^II^ complexes were obtained by allowing copper(II) nitrate to react with the fluorinated **phen** derivatives (*cf*. S‐1). Depending on the crystallization conditions, two different solvates of the complex [Cu(**F_2_phen**)_2_(NO_3_)]NO_3_ were obtained, the solid‐state molecular structures of which were determined by X‐ray diffraction (see Experimental Section). Crystallization from water/ methanol/diethyl ether afforded a dihydrate (**Cu(F_2_phen)_2_(a)**), in which the Cu center is hexa‐coordinated by two bidentate **F_2_phen** ligands, one monodentate nitrate ligand, and one aqua ligand in a typical square‐bipyramidal fashion, with the axial positions being defined by an N atom and the nitrate O atom (Figure 2 a). This arrangement is most likely supported by an intramolecular O−H⋅⋅⋅O bond between the nitrate and aqua ligands. A different picture was observed when crystallization was conducted in the absence of water, affording a mono‐MeOH solvate (**Cu(F_2_phen)_2_(b)**). In this case, the Cu^II^ ion displays a somewhat square‐pyramidal coordination by the two **F_2_phen** ligands and one nitrato ligand, with an N atom defining the apical position (Figure 2 b). However, the structure of the MeOH solvate can be interpreted as an intermediate case between penta‐ and hexa‐coordination, as the sixth coordination site is occupied by a weak, additional Cu⋅⋅⋅O contact to the nitrato ligand. The complex [Cu(**Fphen**)_2_(ONO_2_)]NO_3_, even though crystallizing from wet methanol with one equivalent of crystal water, also displays only a penta‐coordination (*cf*. Figure S‐3.3). However, in this case the arrangement of the ligands is better described as trigonal‐bipyramidal, with two N atoms defining the axial positions. This coordination has been observed most frequently in previously reported [Cu(**phen**)_2_]^2+^ derivatives.[^12^, ^13^, ^14^, ^15^]

**Figure 2:**
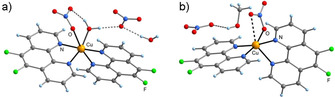
Molecular structures of a) the dihydrate **Cu(F_2_phen)_2_(a)**, and b) the methanol solvate **Cu(F_2_phen)_2_(b)** in the solid state.

The Cu^II^ complexes with fluorinated **phen** ligands showed remarkable changes of half‐wave potentials *E*_1/2_ depending on the substituents of their ligands (Table 1, Figure S‐4). The Cu^II^ complex with two fluorine atoms at the **phen** ligand was the easiest to reduce. As a result, substitution with a strongly electron‐withdrawing group like F or CF_3_ (and even more with two of them) stabilizes the corresponding Cu^I^ species. Similarly, substitution of the **phen** scaffold with CF_3_ groups in positions 2 and 9 led to the highest potential for Cu^II^/Cu^I^ ever measured in a reversible redox process for a mononuclear copper complex (+1.1 V vs. FcH/FcH^+^; FcH=ferrocene).^[16]^ The electron‐withdrawing capability of the substituents (H<F<CF_3_≈SCF_3_<SF_5_)[^17^, ^18^] correlates with the increase in half‐wave potential of the corresponding copper complexes. A similar correlation of the electron‐withdrawing properties of substituents and the reduction potential has been observed before for example, for metalloporphyrins.^[19]^

**Table 1:** Half‐wave potentials *E*_1/2_ of Cu^II^ complexes vs. FcH/FcH^+^ in a 0.1 m KCl solution (water/acetonitrile=9:1) at room temperature.

Complex	*E*_1/2_ [V]	Complex	*E*_1/2_ [V]
[Cu(**phen**)_2_]^2+^	−0.32	[Cu(**CF_3_phen**)_2_]^2+^	−0.21
[Cu(**Fphen**)_2_]^2+^	−0.23	[Cu(**SCF_3_phen**)_2_]^2+^	−0.16
[Cu(**F_2_phen**)_2_]^2+^	−0.11	[Cu(**SF_5_phen**)_2_]^2+^	−0.14

With these trends in mind, the nucleolytic activity of the complexes in the presence and absence of reducing agents towards supercoiled plasmid DNA was investigated. In the presence of ascorbic acid (Figure 3, representative agarose gel Figure S‐5.1) the unmodified [Cu(**phen**)_2_]^2+^ species showed the highest cleavage efficiency of pBR322 DNA followed by the monofluorinated [Cu(**Fphen**)_2_]^2+^ as indicated by formation of linear DNA. The **F_2_phen** and **CF_3_phen** complexes showed nearly equal cleavage efficiency leading to about 80 % open circular and a little amount of linear DNA after 60 min. The sulfur‐containing species [Cu(**SCF_3_phen**)_2_]^2+^ and [Cu(**SF_5_phen**)_2_]^2+^ turned out to be most ineffective nucleases, generating only about 60 % cleaved DNA.

**Figure 3:**
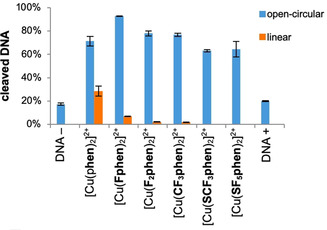
Graphical representation of cleavage of pBR322 DNA (0.025 μg μL^−1^) with different Cu^II^ complexes (10 μm) in the presence of ascorbic acid (250 μm) in 50 mm Tris‐HCl (pH 7.4) after 60 min of incubation at 37 °C. First lane: DNA reference without ascorbic acid. Last lane: DNA reference with ascorbic acid.

The order of activity, [Cu(**phen**)_2_]^2+^>[Cu(**Fphen**)_2_]^2+^>[Cu(**F_2_phen**)_2_]^2+^>[Cu(**CF_3_phen**)_2_]^2+^>[Cu(**SCF_3_phen**)_2_]^2+^≈[Cu(**SF_5_phen**)_2_]^2+^, correlates well with the half‐wave potentials with the exception of the complex with the difluorinated ligand, **F_2_phen**, which showed the least negative *E*_1/2_ value. In order to completely elucidate the nucleolytic activity, not only redox activity, but also affinity to DNA has to be considered. DNA affinity was determined with DNA melting experiments (*cf*. section S‐6) and ethidium bromide (EB) displacement assays with CT‐DNA (*cf*. section S‐7, Table 2).

**Table 2:** Stern–Volmer constants (*K*_SV_) and apparent binding constants (*K*_app_) of different complexes to CT‐DNA (20 μm) derived from EB displacement assay in 10 mM Tris‐HCl.

Complex	*K*_SV_ [m ^−1^]	[Q]_50 %_ [μm]^[a]^	*K*_app_ [m ^−1^]
[Cu(**phen**)_2_]^2+^	2.57×10^4^	38.9	1.29×10^6^
[Cu(**Fphen**)_2_]^2+^	1.73×10^4^	57.8	8.65×10^5^
[Cu(**F_2_phen**)_2_]^2+^	6.68×10^3^	149.7	3.34×10^5^
[Cu(**CF_3_phen**)_2_]^2+^	7.59×10^3^	131.8	3.80×10^5^
[Cu(**SCF_3_phen**)_2_]^2+^	1.03×10^4^	97.1	5.15×10^5^
[Cu(**SF_5_phen**)_2_]^2+^	8.89×10^3^	112.5	4.45×10^5^

[a] Concentration at which 50 % of EB fluorescence is quenched.

The melting temperature *T_m_* of CT‐DNA was increased by 13 °C by the most efficient DNA cleaving agent [Cu(**phen**)_2_]^2+^, whereas for the fluorinated species Δ*T*_m_ of only 1 °C to 8 °C was observed (*cf*. S6). Likewise, EB displacement showed highest affinity, that is, highest *K*_SV_ and *K*_app_ constants (Table 2) for the complexes of highest nucleolytic activity ([Cu(**phen**)_2_]^2+^>[Cu(**Fphen**)_2_]^2+^). However, the affinity of the least active sulfur‐containing species ([Cu(**SCF_3_phen**)_2_]^2+^>[Cu(**SF_5_phen**)_2_]^2+^) was comparable to the one of the more active difluorinated and trifluoromethylated species ([Cu(**CF_3_phen**)_2_]^2+^>[Cu(**F_2_phen**)_2_]^2+^). Small differences in the outcome of the DNA melting and EB displacement studies probably result from the fact that different mechanisms underlie the two methods. Nevertheless, both experiments indicated a dependence of the strength of DNA binding on the steric bulk of the complexes. The larger the substituent (e.g., CF_3_ vs. F) and the higher the grade of substitution (2×F vs. F) on the **phen** scaffold, the lower was the affinity of the resulting complexes to DNA. Accordingly, also a lower nucleolytic activity of the complexes was observed.

Interestingly, in the absence of any added external reducing agents unexpectedly high nucleolytic activity in case of the fluorine‐containing complexes was observed—in contrast to the parent compound (Figure 4, representative agarose gel Figure S‐5.2). [Cu(**F_2_phen**)]^2+^ was 2.5 times more active than [Cu(**phen**)_2_]^2+^, which had an activity barely above the background (DNA reference). When Tris‐HCl was used instead of MOPS as a buffer (Figure S‐5.3), the trends were the same as in MOPS, however, plasmid DNA was even cleaved into its linear form by [Cu(**F_2_phen**)]^2+^. It should be mentioned though that Tris (tris(hydroxymethyl)‐aminomethane), which is commonly used in such assays, is a potential competitive ligand for Cu^II^.^[20]^ According to the literature‐reported association constants log*K* (Table S‐5) also ternary complexes with Tris and **phen** as ligands are conceivable to play a role in the DNA cleavage reaction. (In case of the cleavage reaction in the presence of ascorbate, the buffer change did not reveal any differences.)

**Figure 4:**
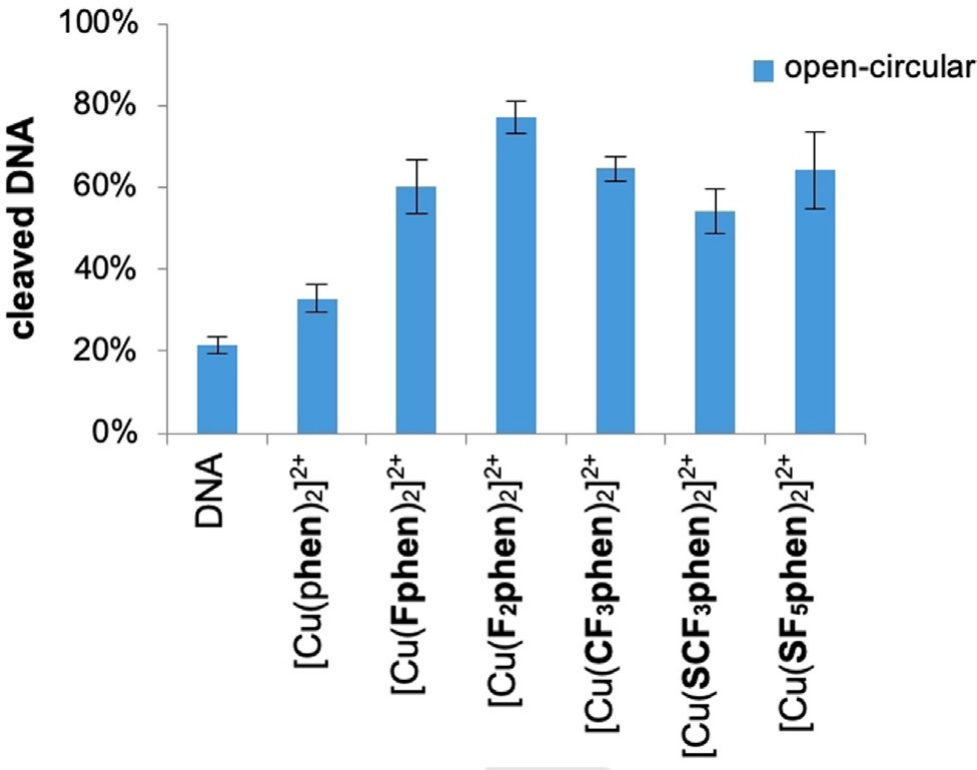
Graphical representation of cleavage of pBR322 DNA (0.025 μg μL^−1^) with different Cu^II^ complexes (100 μm) in 50 mm MOPS (pH 7.4) after 60 min of incubation at 37 °C. First lane: DNA reference.

The nucleolytic activity in the absence of added external reducing agents correlates linearly with the half‐wave potentials *E*_1/2_ of the complexes (Table 1, *cf*. Figure S‐5.5 correlation coefficient *r*=0.86). With the reduction potential becoming less negative the Cu^I^ species is expected to be stabilized by the respective electron‐withdrawing substituents at the **phen** moiety. Such a stabilization apparently leads to higher DNA cleavage rates. As a consequence, a higher degree of fluorination caused a higher activity of the complexes (*cf*. [Cu(**F_2_phen**)_2_]^2+^ vs. [Cu(**Fphen**)_2_]^2+^) in DNA cleavage.

Cleavage without the participation of external reducing agents like ascorbate might indicate a hydrolytic cleavage mechanism, however, also photocleavage or oxidative mechanisms initiated by reducing species in the incubation solution are conceivable. Hydrolytic cleavage was excluded due to demonstrated O_2_‐ and light‐dependence of the cleavage reaction: Under O_2_‐depleted conditions DNA cleavage activity of [Cu(**F_2_phen**)_2_]^2+^ decreased indicating an oxidative mechanism. When the reaction was carried out in the dark, the activity decreased even more (additive effects of light and O_2_) pointing to a photocleavage process (Figure S‐5.4). Whereas the results were not conclusive for the parent compound—probably due to low cleavage activity—a process similar to the one of type I photosensitizers could be proposed for [Cu(**F_2_phen**)_2_]^2+^.^[21]^

Also, there is indication from the literature that photoreduction of Cu^II^ to Cu^I^ in **phen** complexes is possible. In line with what we have observed, the parent compound is photochemically rather inert, but higher activity is expected for species with less negative redox potentials, that is, higher tendency for forming Cu^I^.^[22]^ Such Cu^I^ species are prone to ROS generation.^[23]^

Alternatively, DNA itself could serve as a reducing agent. DNA bases like guanine exhibit a relatively low oxidation potential,^[24]^ especially when considering Watson–Crick G⋅C base pairing.[^25^, ^26^] A pathway involving Cu^III^ species like in the case of Cu^II^ hydroxysalene complexes^[27]^ is improbable due to the electron‐withdrawing property of the fluorine‐containing functional groups.^[28]^

To test for ROS involved in the cleavage reaction scavenging experiments were carried out. The quenching of cleavage activity by catalase (cat.) and superoxide dismutase (SOD) showed the participation of hydrogen peroxide and superoxide (Figure 5). No quenching was observed in case of DMSO and NaN_3_ which makes it unlikely that freely diffusible hydroxyl radicals or singlet oxygen are involved as ROS. It seems that the formation of ROS proceeds in a similar manner like the formation of ROS in the presence of external reducing agents like ascorbate including the stepwise one‐electron reduction of oxygen to superoxide and furthermore to a peroxo species.^[29]^

**Figure 5:**
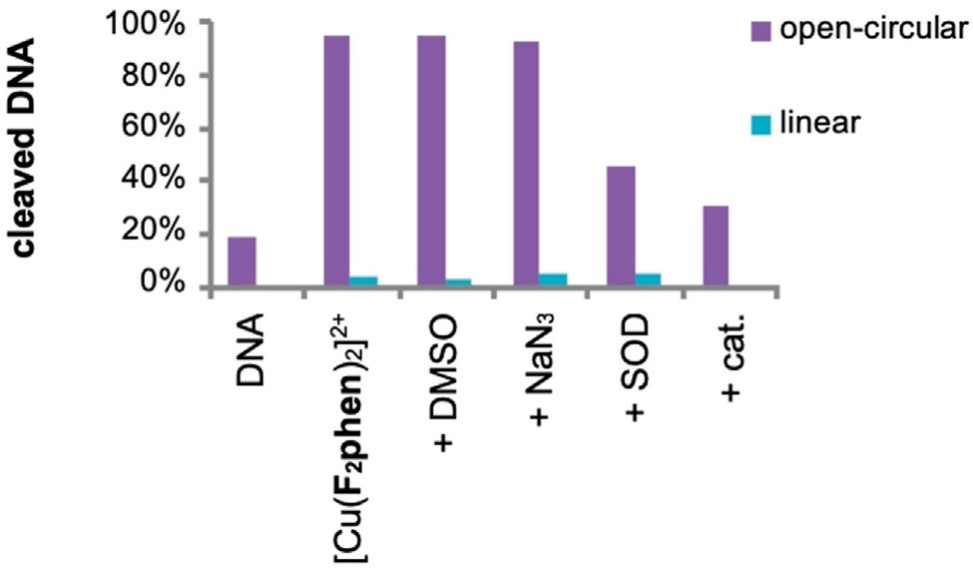
Cleavage of pBR322 DNA (0.025 μg μL^−1^) with [Cu(**F_2_phen**)_2_]^2+^ (100 μm) in the presence of different ROS scavengers (detailed conditions in the Supporting Information) in 50 mm MOPS (pH 7.4) after 60 min of incubation at 37 °C. First lane: DNA reference without complex and ROS scavengers. Second lane: reference without ROS scavengers.

In order to evaluate the influence of fluorine‐containing substituents on cytotoxicity, the MTT assay was carried out with MCF‐7 breast cancer cells. Figure 6 shows cell viability in the presence of 10 μm Cu^II^ complexes with fluorinated ligands in comparison to [Cu(**phen**)_2_]^2+^ with unsubstituted **phen**.

**Figure 6:**
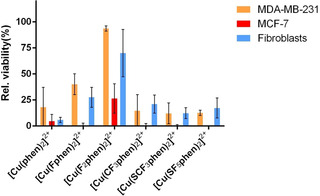
Cytotoxicity of different complexes (10 μm) to MDA‐MB‐231 and MCF‐7 breast cancer cells as well as fibroblasts resulting from an MTT assay after 48 h of incubation.

[Cu(**CF_3_phen**)_2_]^2+^, [Cu(**SCF_3_phen**)_2_]^2+^ and [Cu(**SF_5_phen**)_2_]^2+^ showed nearly the same cytotoxicity as the parent compound [Cu(**phen**)_2_]^2+^ in MCF‐7 cells (IC_50_ 2.2–2.7 μm vs. 2.3 μm, Figure S‐8, Table S‐8), whereas [Cu(**Fphen**)_2_]^2+^ and even more [Cu(**F_2_phen**)_2_]^2+^ distinctly exhibited a much lower cytotoxicity with IC_50_ values being two to three times higher. These trends were also observed in the more aggressive breast cancer cells MDA‐MB‐231^[30]^ and in fibroblasts, the latter ones representing healthy cells. The cytotoxicity was lower for all compounds in these cell lines, whereby the fluorinated compounds were in general clearly less cytotoxic towards fibroblasts than cancer cells when compared to [Cu(**phen**)_2_]^2+^. As an example, the IC_50_ value of [Cu(**F_2_phen**)_2_]^2+^ incubated with fibroblasts was twice as high as the one in cancer cells indicating that it has a slightly reduced cytotoxic effect towards non‐cancer cells.

There is no correlation between the cytotoxicity and nucleolytic activity and DNA affinity of the complexes so that also other mechanisms have to be considered in inducing cell death. Indeed, lipophilicity seems to be decisive here to explain the cytotoxicity trends for the fluorinated compounds: CF_3_, SCF_3_ and SF_5_ show higher lipophilicity increments in comparison to F,^[31]^ which was confirmed for the complexes through the water/*n*‐octanol partition experiment[^32^, ^33^] (log *P* for complexes with **phen** substituents H<F<2×F<CF_3_<SCF_3_<SF_5_, Table S‐9). The compounds with the latter substituents (CF_3_, SCF_3_, SF_5_) showed higher (less negative) log *P* values (higher lipophilicity) than the ones with the first ones (F, 2×F) which correlates well with the observed cytotoxic behavior. The fact that the parent compound representing the least lipophilic complex, [Cu(**phen**)_2_]^2+^, is similarly cytotoxic as the most lipophilic ones, confirms that lipophilicity is only one factor determining cytotoxicity.

It has to be mentioned that recent findings indicate that Cu^II^ **phen** complexes, although stable at pH 7.4 and 37 °C in water (as demonstrated in Figure S‐10 by UV/VIS spectroscopy) most probably decompose in cell culture media. Interaction with cell components and cell death may be due to Cu ions and **phen** acting separately.^[34]^ Also, thiol‐rich molecules such as metallothioneins and glutathione, abundant in the cytosol and nucleus, might reduce Cu^II^ and scavenge reduced Cu^I^ even before the complex reaches its potential target, the DNA.^[35]^ Such behavior is even more probable for longer incubation times (>24 h). However, in these reported studies the amount of Cu accumulated in cell compartments was different for different **phen** derivatives, indicating that efficiency of uptake, and eventually also cell death, indeed depends on the ligand moiety.^[34]^

In conclusion, we have synthesized Cu^II^ complexes of the type [Cu(**phen**)_2_]^2+^ with fluorinated **phen** ligands, which act as chemical nucleases without the otherwise required classical, bioabundant external reducing agents. This modification shows a high value for the construction of such nucleases via simple derivatization of ligands, changing the electronic properties of the [Cu(**phen**)_2_]^2+^ complex. Substitution of the ligands with fluorine‐containing groups is responsible for a less negative reduction potential of the complexes thus enabling an activation of the nuclease without external reducing agent. All complexes showed high cytotoxicity in two different breast cancer cell lines (IC_50_<10 μm), and the more lipophilic the ligand, the higher the cytotoxic effect was. Among the fluorinated complexes, despite of being the least cytotoxic compound, [Cu(**F_2_phen**)_2_]^2+^ stood out by showing the most distinctive differentiation between cancer and healthy cells. This complex was also the most efficient DNA cleaver outperforming the parent compound [Cu(**phen**)_2_]^2+^, which does need an external reducing agent like ascorbate for initiation of its nucleolytic activity. It will be of interest to further investigate the mechanism of activation of nucleolytic activity.

## Experimental Section

For the synthesis of complexes, methods and molecular and cell biological studies see the Supporting Information.

Human dermal fibroblasts isolated from neonate foreskin biopsies after ethical approval (EA1/081/13, Ethics Committee from the Charité Campus Mitte, Berlin) and with informed parental consent, were provided by the Institute of Pharmacy (Freie Universität Berlin).

Deposition numbers 2032216 (**Cu(F_2_phen)_2_(a)**) and 2032217 (**Cu(F_2_phen)_2_(b)**) contain the supplementary crystallographic data for this paper. These data are provided free of charge by the joint Cambridge Crystallographic Data Centre and Fachinformationszentrum Karlsruhe Access Structures service.

## Conflict of interest

The authors declare no conflict of interest.

## Supporting information

As a service to our authors and readers, this journal provides supporting information supplied by the authors. Such materials are peer reviewed and may be re‐organized for online delivery, but are not copy‐edited or typeset. Technical support issues arising from supporting information (other than missing files) should be addressed to the authors.

Supplementary
