# Supplementary material for: Forty Years after the Discovery of Its Nucleolytic Activity: [Cu(phen)2]2+ Shows Unattended DNA Cleavage Activity upon Fluorination
Source: Chemistry. 2021 Jan 22;27(10):3273–7. doi: 10.1002/chem.202004594 (PMC7898652; doi:10.1002/chem.202004594)
Supplement: Supplementary file 1 — Supplementary [file CHEM-27-3273-s001.pdf]

Chemistry–A European Journal

Supporting Information

Forty Years after the Discovery of Its Nucleolytic Activity: $[Cu(phen)_2]^{2+}$ Shows Unattended DNA Cleavage Activity upon Fluorination

Carsten Lüdtke,[a] Sebastian Sobottka,[a] Julian Heinrich,[b] Phil Liebing,[b] Stefanie Wedepohl,[c] Biprajit Sarkar,[a, d] and Nora Kulak*[a, b]

Electronic Supplementary Information

Accompanying the manuscript

Forty years after the discovery of its nucleolytic activity: $[Cu(phen)_2]^{2+}$ shows unattended DNA cleavage activity upon fluorination

Carsten Lüdtke, Sebastian Sobottka, Julian Heinrich, Phil Liebing, Stefanie Wedepohl, Biprajit Sarkar, and Nora Kulak*

List of contents:

S-1 Syntheses
S-2 Methods
S-3 X-ray crystallography
S-4 Cyclic voltammetry
S-5 Gel electrophoresis
S-6 DNA melting curves
S-7 Ethidium bromide displacement
S-8 MTT assay
S-9 Determination of the partition coefficient log *P*
S-10 Stability tests
References

S-1 Syntheses

S-1.1 General considerations

All reactions were performed under atmospheric conditions without exclusion of air. The fluorinated **phen** derivatives were synthesized according to literature previously published by our group.[1] [Cu(**phen**)$_2$(H_2O)](NO_3)$_2$ was prepared as reported previously.[2] All other starting materials and solvents were obtained from commercial suppliers and used without further purification.

S-1.2 Preparation of the complexes

Synthesis of [Cu(Fphen)$_2$(NO$_3$)]NO$_3$: 5-Fluoro-1,10-phenanthroline (**Fphen**, 198 mg, 1.0 mmol) and Cu(NO_3)$_2$·3H_2O (120 mg, 0.5 mmol) were dissolved in 5 mL methanol each. The copper(II) salt solution was added dropwise to the **Fphen** solution and heated to reflux for 60 min. After cooling to ambient temperature, the precipitated product was isolated by filtration, washed with cold methanol and the turquoise solid was dried *in vacuo*. Yield: 215 mg (0.37 mmol; 74%). Anal. calcd. for $C_{24}H_{14}F_2N_6O_6Cu$: C 49.36; N, 14.39; H, 2.42 %, Found: C, 49.21; N, 14.17; H, 2.47 %; max. deviation [%] 0.22. ESI-MS: Calcd. for [Cu(**Fphen**)$_2$]$^+$ 459.048, found 459.056. UV/VIS (H_2O): λ_{max} (ε) = 699 nm (475 L mol^{-1} cm^{-1}). Single crystals suitable for X-ray structure elucidation were obtained by slow evaporation of a methanol solution at ambient temperature.

Synthesis of [Cu(CF$_3$phen)$_2$(NO$_3$)]NO$_3$: 5-(Trifluoromethyl)-1,10-phenanthroline (**CF$_3$phen**, 248 mg, 1.0 mmol) and Cu(NO_3)$_2$·3H_2O (120 mg, 0.5 mmol) were dissolved in 1 mL methanol each. The copper(II) salt solution was added dropwise to the **CF$_3$phen** solution and heated to reflux for 20 min. After cooling to ambient temperature, the solvent was removed under reduced pressure. Some drops of water were added to the remaining residue, which was then dissolved in 5 mL THF. This solution was stored at –25 °C for two days. The formed precipitate was filtered off rapidly in the cold, thoroughly washed with cold (< –20 °C) *n*-hexane, and the blue solid was dried *in vacuo*. Yield: 150 mg (0.19 mmol; 38%). Anal. calcd. for $C_{30}H_{25}F_6N_6O_{8.5}Cu$ (≡ [Cu(**CF$_3$phen**)$_2$(NO_3)]NO_3·1.5H_2O·THF): C, 46.01; N, 10.73; H, 3.22 %, Found: C, 46.13; N, 10.94; H, 3.15 %; max. deviation [%] 0.21. ESI-MS: Calcd. for [Cu(**CF$_3$phen**)$_2$]$^+$ 559.042, found 559.036. UV/VIS (H_2O): λ_{max} (ε) = 697 nm (595 L mol^{-1} cm^{-1}).

Synthesis of [Cu(SCF$_3$phen)$_2$(NO$_3$)]NO$_3$: 5-(Trifluoromethylthio)-1,10-phenanthroline (**SCF$_3$phen**, 100 mg, 0.36 mmol) and Cu(NO_3)$_2$·3H_2O (41 mg, 0.17 mmol) were dissolved in 1 mL methanol each. The copper(II) salt solution was added dropwise to the **SCF$_3$phen** solution and heated to reflux for 20 min. After cooling to ambient temperature, the solvent was removed under reduced pressure. Some drops of water were added to the residue, which was then dissolved in 5 mL THF. This solution was stored at −25 °C for two days. The formed precipitate was filtered off rapidly in the cold, thoroughly washed with cold (< −20 °C) *n*-hexane, and the blue solid dried *in vacuo*. The crude product was recrystallized by dissolving in 1 mL ethanol, addition of some drops of water, and subsequent slow diffusion of diethyl ether into the solution. Yield: 45 mg (0.058 mmol, 34%). Anal. calcd. for $C_{27}H_{17}F_6N_6O_{6.5}S_2Cu$ (= [Cu(**SCF$_3$phen**)$_2$(NO_3)]NO_3·0.5EtOH): C, 42.06; N, 10.90; H, 2.22; S, 8.32 %, Found: C, 41.79; N, 11.22; H, 2.54; S, 8.61 %; max. deviation [%] 0.32. ESI-MS: Calcd. for [Cu(**SCF$_3$phen**)$_2$]$^+$ 622.986, found 622.985. UV/VIS (H_2O): λ_{max} (ε) = 705 nm (541 L mol^{-1} cm^{-1}).

Synthesis of [Cu(SF_5phen)$_2$(NO_3)]NO_3: 5-(Pentafluorosulfanyl)-1,10-phenanthroline (**SF_5phen**, 58 mg, 0.19 mmol) and $Cu(NO_3)_2·3H_2O$ (22 mg, 0.09 mmol) were dissolved in 0.5 mL methanol each. The copper(II) salt solution was added dropwise to the **SF_5phen** solution and heated to reflux for 20 min. After cooling to ambient temperature, the solvent was removed under reduced pressure. Some drops of water were added to the residue, which was then dissolved in 5 mL THF. This solution was stored at –25 °C for two days. The formed precipitate was filtered off rapidly in the cold, thoroughly washed with cold (< –20 °C) *n*-hexane, and the blue solid was dried *in vacuo*. The crude product was recrystallized by dissolving in 1 mL *n*-propanol and subsequent diffusion of diethyl ether into the solution. Yield: 20 mg (0.024 mmol, 27%). Anal. calcd. for $C_{25}H_{18}F_{10}N_6O_7S_2Cu$ (≡ [Cu(**SF_5phen**)$_2$(NO_3)]NO_3·MeOH): C, 36.09; N, 10.10; H, 2.18; S, 7.71 %, Found: C, 36.05; N, 10.43; H, 2.55; S, 7.99 %; max. deviation [%] 0.37. ESI-MS: Calcd. for [Cu(**SF_5phen**)$_2$]$^+$ 674.980, found 674.973. UV/VIS (H_2O): λ_{max} (ε) = 701 nm (535 L mol^{-1} cm^{-1}).

Synthesis of [Cu(F_2phen)$_2$$NO_3$]$NO_3$: 5,6-Difluoro-1,10-phenanthroline (**F_2phen**, 216 mg, 1.0 mmol) and $Cu(NO_3)_2·3H_2O$ (120 mg, 0.5 mmol) were dissolved in 5 mL methanol each. The copper(II) salt solution was added dropwise to the **F_2phen** solution and heated to reflux for 60 min. After cooling to ambient temperature, a blue precipitate was obtained, which was filtered off, washed with cold methanol and dried *in vacuo*. Yield: 260 mg (0.40 mmol, 80%). Anal. calcd. for $C_{24.5}H_{14}F_4N_6O_{6.5}Cu$ (≡ [Cu(**F_2phen**)$_2$(NO_3)]NO_3·0.5 MeOH): C, 46.27; N, 13.21; H, 2.22 %, Found: C, 46.46; N, 13.22; H, 2.44 %; max. deviation [%] 0.22. ESI-MS: Calcd. for [Cu(**F_2phen**)$_2$]$^+$ 495.029, found 495.035. UV/VIS (H_2O): λ_{max} (ε) = 695 nm (403 L mol^{-1} cm^{-1}). Single crystals suitable for X-ray structure elucidation were obtained by slow diffusion of diethyl ether into a methanol solution ([Cu(**F_2phen**)$_2$(NO_3)]NO_3·MeOH; **Cu(F_2phen)$_2$(a)**), or into a methanol/water solution ([Cu(**F_2phen**)$_2$(NO_3)]NO_3·2H_2O; **Cu(F_2phen)$_2$(b)**).

S-2 Methods

Elemental analysis: Elemental analyses were carried out with a Elementar Vario EL (Elementar Analysensysteme GmbH) with two columns for sulfur-free and three columns for sulfur-containing samples, respectively.

Mass spectrometry: ESI mass spectra were measured on an Agilent 6210 ESI-ToF mass spectrometer. The flow rate was 4 µL/min and the spray voltage 4 kV. The pressure of the desolvation gas was set to 1 bar and the other parameters were adjusted for the maximum abundance of the respective $[CuL_2]^{+/2+}$ molecular ion peak. The samples were dissolved in acetonitrile, methanol or a mixture of these solvents.

X-Ray crystallography: Single-crystal X-ray data were collected on a Bruker Apex II diffractometer equipped with an Apex II CCD detector, using graphite-monochromated Mo–K_{α} radiation. Absorption correction of the intensity data was carried out with the multi-scan method.[3] The structures were solved with ShelXS-2014/7[4] with direct methods, and refined by full matrix least-squares methods on F^2 (SHELXL-2014/7)[5] in OLEX2[6].

UV/VIS spectroscopy: Absorbance measurements, stability tests of the complexes, DNA melting studies and determination of the partition coefficient log *P* were carried out in 10 mm quartz cuvettes (Hellma Analytics) using an Agilent Cary 100 Bio UV/VIS spectrophotometer.

DNA melting: DNA melting curves were measured in Tris-HCl buffer (10 mM, pH 7.4) in a temperature range 50-97 °C applying a heating rate of 1 °C/min. The absorption was measured every 30 s. For all experiments the CT-DNA:complex ratio was 10:1 (250 µM:25 µM). The concentration of CT-DNA was calculated using $\varepsilon = 6600\ M^{-1}\ cm^{-1}$ as the extinction coefficient.[7] The melting curves were normalized (A_{max}=1, A_{min}=0) and the melting temperature T_M was determined at A=0.5. All experiments were carried out in duplicate.

Determination of the partition coefficient (log *P*): Log *P* determination, where *P* is the water-*n*-octanol partition coefficient, was adapted from Bonnet *et al.*[8] and Khnayzer *et al.*[9] with a slightly different procedure protocol while taking into consideration the EPA guidelines (EPA 712–C–96–038).
To 1 mL of a 0.75 mM complex solution in *n*-octanol-saturated water were pipetted 1 mL water-saturated *n*-octanol in a 2 mL Eppendorf tube. The Eppendorf tubes were shaken by hand every 10 min for 1 h at room temperature. The samples were centrifuged for 10 min at approx. 6000 rpm with a FisherBrand SPROUT Mini Centrifuge. Two phases were formed: An upper *n*-octanol and a lower water phase. The upper octanol phase was decanted off. 750 µL of the lower water phase was carefully pipetted into a cuvette. The Cu(II) complex concentration in the water phase was determined photometrically through the corresponding absorption coefficient ε at λ_{max} of the d-d transition (*cf.* S-1.2, for $[Cu(\mathbf{phen})_2]^{2+}$ λ_{max} (ε) = 704 nm (576 L mol^{-1} cm^{-1}) was determined). The experiments were carried out in duplicate and one sample contained no complex, where the water phase was applied as baseline in the UV/VIS absorption measurement. The following equation was used to calculate the log *P* values which are listed in Table S-9.

$$\log P = \log \frac{[complex]_{octanol}}{[complex]_{aq}} = \log \frac{[complex]_{total} - [complex]_{aq}}{[complex]_{aq}}$$

Cyclic voltammetry: Electrochemical measurements were carried out in 9:1 water (Millipore):acetonitrile ($H_2O \leq 0.01$ %, puriss., Sigma Aldrich, distilled over CaH_2 before usage) with 0.1 M KCl as a supporting electrolyte under nitrogen atmosphere (Nitrogen 5.0) at room temperature. A three-electrode setup (working electrode: glassy carbon; counter electrode: platinum wire; pseudoreference electrode: silver wire) and a PAR VersaStat 4 potentiostat (Ametek) with the VersaStudio software was used. As internal standards, 1,4-benzoquinone and ferrocene were applied (*i.e.* 1,4-benzoquinone was used in the experiment due to its higher solubility in water:acetonitrile 9:1, and the values were then converted *vs.* FcH/FcH^+ based on a separate measurement of 1,4-benzoquinone and ferrocene in that solvent).[10] All measurements were referenced against the ferrocene/ferrocenium redox couple. As scan rates, 25, 50, 100, 250 und 500 mV s^{-1} were chosen.

Ethidium bromide displacement: The fluorescence emission spectra of intercalated ethidium bromide (EB) were collected using a Varian Cary Eclipse fluorescence spectrophotometer. The excitation wavelength was 518 nm and the emission spectra were recorded in the range from 530 to 750 nm (scan rate 120 nm/min, data interval 1 nm). In 10 mm cuvettes (Hellma Analytics), first a spectrum of EB (5 µM) in Tris-HCl buffer (10 mM) in the presence and absence of CT-DNA (25 µM) was recorded. The respective complex was subsequently titrated into the cuvette such that the added volume did not exceed 15 µL and the total volume of the solution of 1 mL did not significantly change. The voltage of the photomultiplier was adjusted to 900 V in order to keep the emission of the CT-DNA-EB system between 700 and 1000 a.u.
The EB displacement data were evaluated by using the Stern-Volmer equation (1) where I_0 is the fluorescence emission in absence and I is the fluorescence emission at a defined concentration of the competitive molecule [Q]. The Stern-Volmer constant K_{SV} was hereby determined from the slope of the linear regression of a plot of [Q] against I_0/I. The apparent binding constant K_{app} can then be determined from equation (2), using the binding constant K_{EB} of EB towards DNA (10^7 M^{-1})[11], the used concentration [EB] (5 µM), and the concentration of the competitor $[Q]_{50}$, at which 50% of the fluorescence emission of EB has been quenched.

$$\frac{I_0}{I} = 1 + K_{SV}[\mathrm{Q}] \qquad (1)$$

$$K_{EB}[\mathrm{EB}] = K_{app}[\mathrm{Q}]_{50} \qquad (2)$$

Gel electrophoresis: The complexes (concentrations given in the captions of Figures 2-5) were incubated in Tris-HCl buffer (50 mM, pH 7.4) or MOPS buffer (50 mM, pH 7.4) in the presence or absence of ascorbic acid (250 µM) with 0.2 µg plasmid DNA (pBR322) in Eppendorf tubes for 1 h at 500 rpm and 37 °C. The total volume of the incubation solution was adjusted to 8 µL.
For analysis, 1.5 µL of loading buffer (containing 3.7 mM bromophenol blue, 1.2 M sucrose in deionized water) was added to the incubation solution. The sample was loaded onto an agarose (SeaKem LE, Lonza) gel (1% in 0.5X Tris-borate-EDTA (TBE) buffer, Fisher

Scientific) containing ethidium bromide (0.2 μg mL^{-1}, Fisher Scientific). Electrophoresis was carried out at 40 V for 2 h with an electrophoresis unit (Carl Roth; power supply: consort EV243) in 0.5X TBE buffer. Bands were visualized by UV light, photographed by using a gel documentation system (GelDoc, Bio-Rad) and analyzed with the software Image Lab 5.0. For supercoiled DNA a correction factor of 1.22 was used due to the weaker binding of EB to this form of plasmid DNA in comparison to the linear and open-circular form.[12]

For investigating potential cleavage mechanisms, the cleavage reactions were carried out as described above, but the Eppendorf tubes were flushed with argon, and argon was bubbled trough the stock solutions to provide oxygen-depleted conditions. For reactions in the dark, the Eppendorf tubes were wrapped in aluminum foil.

For the quenching experiments, the procedure was the same. However, before incubation, the following ROS scavenging agents were added: (DMSO: 200 mM, NaN_3: 10 mM, SOD: 313 U mL^{-1}, catalase (cat.): 2.5 mg mL^{-1}). Catalase was pre-incubated for 30 min in 1X PBS buffer. All other samples in this experiment were thus also incubated in the presence of PBS (final concentration 0.125X) for ensuring comparability.

All experiments were carried out two to four times, the quenching experiments only once.

Cell culture: MCF-7 cells (DSMZ-Deutsche Sammlung von Mikroorganismen und Zellkulturen GmbH) were routinely maintained in RPMI media without phenol red (Life Technologies) with 10% FBS (fetal bovine serum, Biochrom AG), 1% penicillin/streptomycin (Life Technologies) and 1% MEM (minimal essential medium) non-essential amino acids (PAA Laboratories) at 37 °C and 5% CO_2. MDA-MB-231 cells were purchased from Sigma-Aldrich and cultivated in L-15 medium (Leibovitz) supplemented with 2 mM glutamine, 15% FBS and 1% penicillin/streptomycin. Human dermal fibroblasts isolated from neonate foreskin biopsies[13] after ethical approval (EA1/081/13, Ethics Committee from the Charité Campus Mitte, Berlin) and with informed parental consent, were kindly provided by the Institute of Pharmacy (Freie Universität Berlin). Fibroblasts were cultivated in DMEM supplemented with 10% FBS and 1% penicillin/streptomycin.

MTT assay: 10^5 cells/mL were seeded into 96 well plates at 100 μL/well and incubated overnight at 37 °C and 5% CO_2. The next day, medium was replaced with 50 μL fresh medium and 50 μL dilutions of the test compounds (in duplicate). After 48 h of incubation the medium was removed and replaced by 100 μL fresh medium and 10 μL MTT (3-(4,5-dimethylthiazol-2-yl)-2,5-diphenyltetrazolium bromide, Sigma Aldrich, 5 mg/mL stock solution in PBS). The cells were then incubated another 4 h at 37 °C. The supernatant was discarded and formazan crystals were solubilized in 100 μL/well isopropanol with 0.04 M HCl. Absorbance was read in a Tecan Infinite M200 Pro plate reader at 570 nm. Assays were repeated three times independently.

Relative cell viabilities were calculated by dividing the average absorbance values of wells containing treated cells by the average absorbance value of wells with untreated cells. IC_{50} values were determined by fitting of a non-linear dose-response curve (log inhibitor vs. normalized response, variable slope) using GraphPad Prism software.

S-3 X-ray crystallography

Table S-3. Crystal data and details on structure refinement for the reported compounds.

Compound	[Cu(**F_2phen**)$_2$(H_2O)]NO_3·H_2O {**Cu(F_2phen)$_2$(b)**}	[Cu(**F_2phen**)$_2$(NO_3)]NO_3·MeOH {**Cu(F_2phen)$_2$(a)**}
CCDC deposition number	2032216	2032217
Molecular formula sum	$C_{24}H_{16}CuF_4N_6O_8$	$C_{25}H_{16}CuF_4N_6O_7$
Formula weight / g mol^{-1}	655.97	651.98
Crystal system	monoclinic	monoclinic
Space group	C2/c	$P2_1/n$
Cell metric a / Å	18.4434(9)	7.4038(6)
b / Å	25.709(1)	25.928(2)
c / Å	13.6944(7)	13.607(1)
α / deg.	90	90
β / deg.	130.790(1)	104.900(3)
γ / deg.	90	90
Cell volume / Å^3	4916.2(4)	2524.2(4)
Molecules per cell z	8	4
Electrons per cell F_{000}	2648	1316
Calcd. density ρ / g cm^{-3}	1.773	1.716
μ / mm^{-1} (Mo-K$_\alpha$)	0.984	0.955
Crystal shape and color	light blue needle	blue block
Crystal size / mm	0.59×0.40×0.15	0.32×0.28×0.18
T / K	99(2)	100(2)
θ range / deg.	2.524 ... 26.454	2.206 ... 25.398
Reflections collected	51110	31885
Reflections unique	5060	4633
Reflections with I>2σ(I)	4506	4207
Completeness of dataset	99.9%	99.7%
R_{int}	0.0365	0.0323
Parameters; Restraints	393; 0	391; 0
R_1 (all data, I>2σ(I))	0.0375; 0.0322	0.0331; 0.0283
wR_2 (all data, I>2σ(I))	0.0823; 0.0796	0.0687; 0.0667
GooF (F^2)	1.042	1.066
Max. residual peaks	−0.387; 0.661	−0.371; 0434
Extinction coefficient	0.00040(8)	0.0031(3)

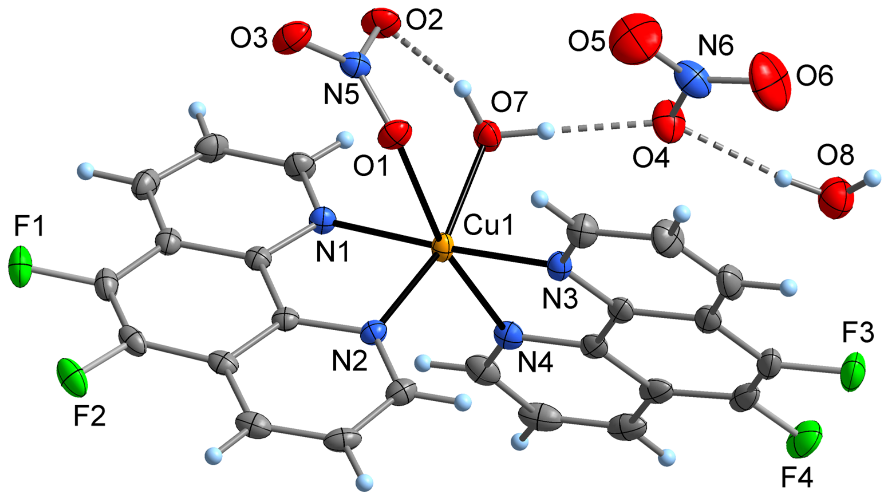

Figure S-3.1. Molecular structure of [Cu(**F₂phen**)₂(H₂O)]NO₃·H₂O {**Cu(F₂phen)₂(a)**} in the crystal, showing the atom numbering scheme. Displacement ellipsoids are drawn at the 50% probability level, hydrogen atoms as spheres of arbitrary size. Selected interatomic distances (pm) and angles (deg.): Cu1-O1 262.9(2), Cu1-O7 201.1(2), Cu1-N1 199.6(2), Cu1-N2 203.5(2), Cu1-N3 200.6(2), Cu1-N4 222.1(2), O1-Cu1-O7 81.21(6), O1-Cu1-N1 82.41(6), O1-Cu1-N2 82.25(6), O1-Cu-N3 90.97(6), O1-Cu1-N4 169.21(6), O7-Cu1-N1 89.06(6), O7-Cu1-N2 162.20(6), O7-Cu1-N3 90.73(6), O7-Cu1-N4 94.15(6), N1-Cu1-N2 82.29(7), N1-Cu1-N3 173.33(7), N1-Cu1-N4 107.36(7), N2-Cu1-N3 96.08(7), N2-Cu1-N4 103.26(6), N3-Cu1-N4 79.30(7), O2···O7 271.4(3), O4···O7 275.8(2), O4···O8 288.1(3), O8···F1' 334.8(4).

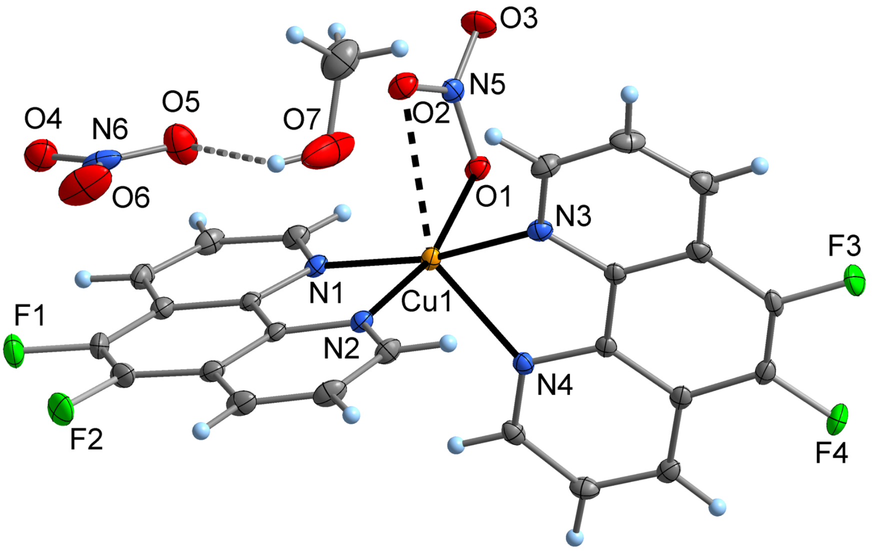

Figure S-3.2. Molecular structure of [Cu(**F₂phen**)₂(NO₃)]NO₃·MeOH {**Cu(F₂phen)₂(b)**} in the crystal, showing the atom numbering scheme. Displacement ellipsoids are drawn at the 50% probability level, hydrogen atoms as spheres of arbitrary size. Selected interatomic distances (pm) and angles (deg.): Cu1-O1 201.9(1), Cu1···O2 262.7(1), Cu1-N1 199.2(2), Cu1-N2 200.0(2), Cu1-N3 202.2(2), Cu1-N4 221.2(2), O1-Cu1···O2 53.97(5), O1-Cu1-N1 90.76(6), O1-Cu1-N2 164.50(6), O1-Cu1-N3 86.27(6), O1-Cu1-N4 93.01(6), N1-Cu1-N2 82.94(7), N1-Cu1-N3 170.10(7), N1-Cu1-N4 110.57(6), N2-Cu1-N3 97.54(7), N2-Cu1-N4 102.46(6), N3-Cu1-N4 79.05(6), O5···O7 281.6(3).

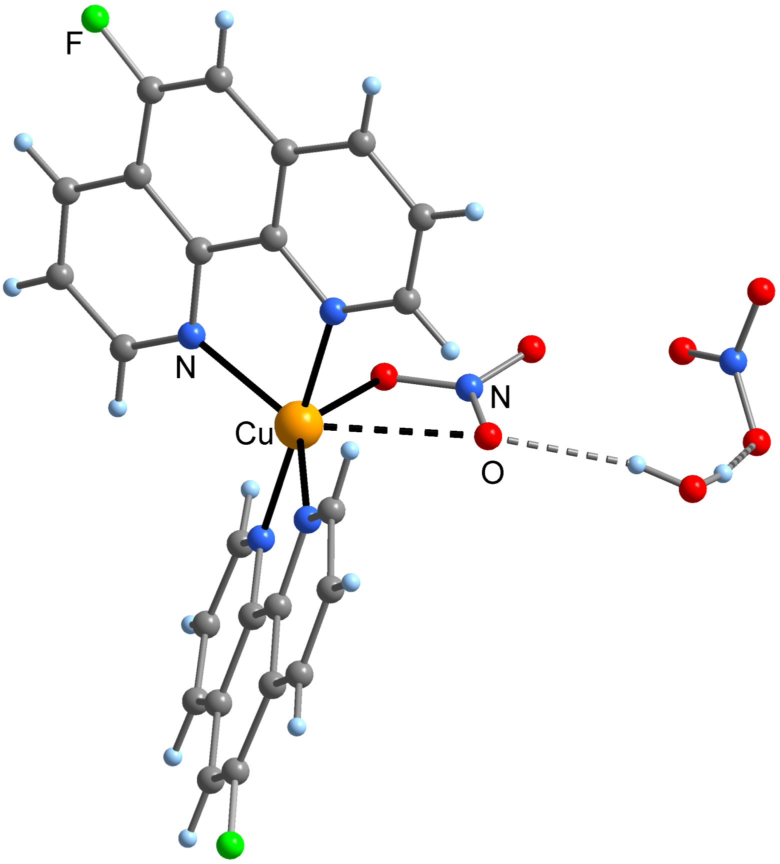

Figure S-3.3. Molecular structure of [Cu(**Fphen**)$_2$(NO_3)]NO_3·H_2O in the crystal. All atoms drawn as spheres of arbitrary size. The crystal quality of this compound did not allow for full structure refinement.

S-4 Cyclic voltammetry

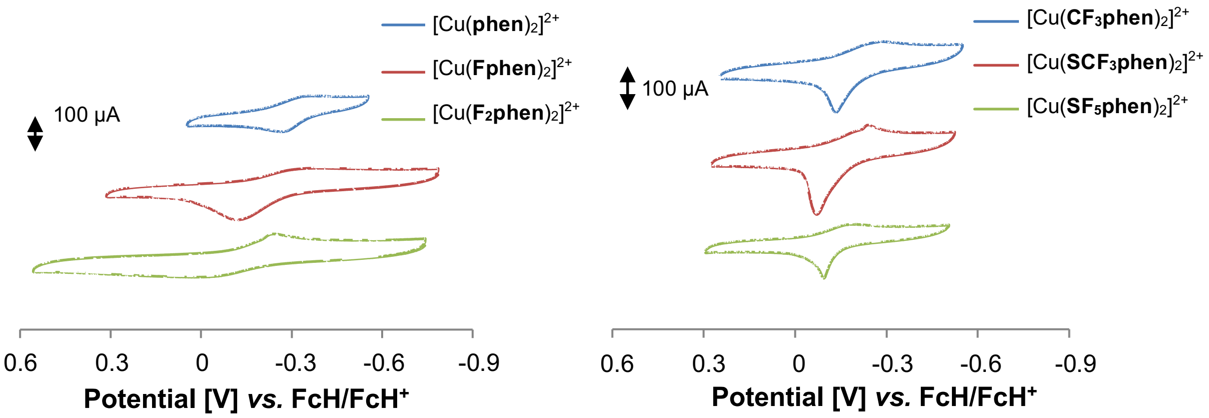

Figure S-4. Cyclic voltammograms of complexes $[Cu(\mathbf{Xphen})_2]^{2+}$ (X = H, F, 2xF, CF_3, SCF_3, SF_5) in a 0.1 M KCl solution (water/acetonitrile=9:1) at room temperature with a scan rate 100 mV s^{-1} referenced *vs.* ferrocene/ferrocenium ($E_{1/2}$ = 0 V).

S-5 Gel electrophoresis

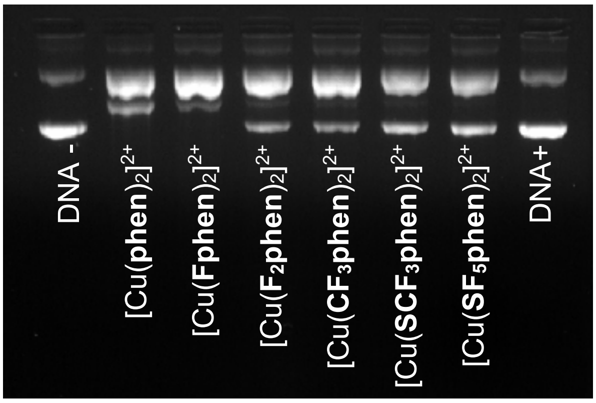

Figure S-5.1. Representative gel for the bar diagram in Figure 3 (pBR322 plasmid DNA (0.025 µg μL^{-1}) with different Cu(II) complexes (10 µM) in the presence of ascorbic acid (250 µM) in 50 mM Tris-HCl (pH 7.4) after 60 min incubation at 37 °C). First lane: DNA reference without ascorbic acid. Last lane: DNA reference with ascorbic acid.

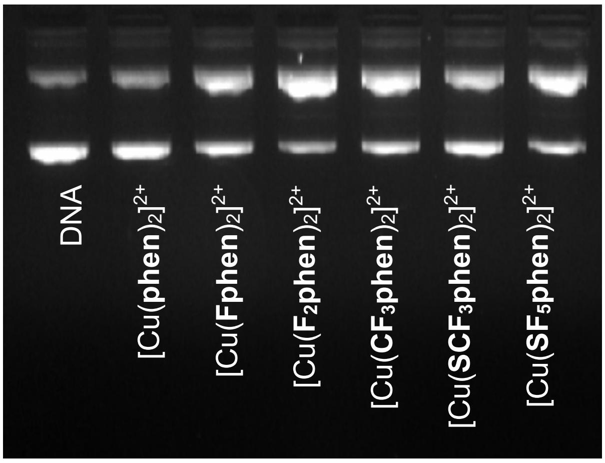

Figure S-5.2. Representative gel for the bar diagram in Figure 4 (pBR322 plasmid DNA (0.025 µg μL^{-1}) with different Cu(II) complexes (100 µM) in 50 mM MOPS (pH 7.4) after 60 min incubation at 37 °C). First lane: DNA reference.

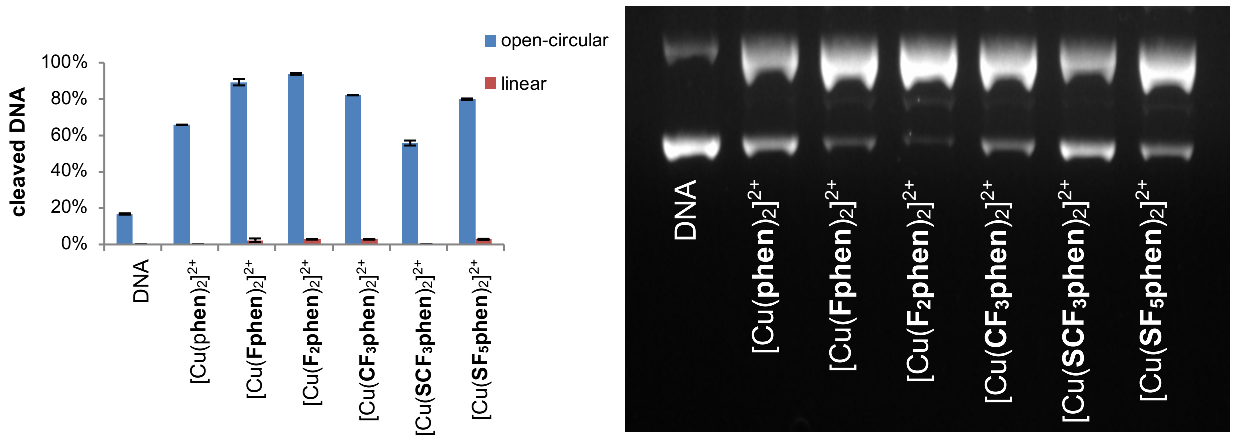

Figure S-5.3. Graphical representation of cleavage and representative gel of pBR322 plasmid DNA (0.025 µg µL^{-1}) with different Cu(II) complexes (100 µM) in 50 mM Tris-HCl (pH 7.4) after 60 min incubation at 37 °C. First lane: DNA reference.

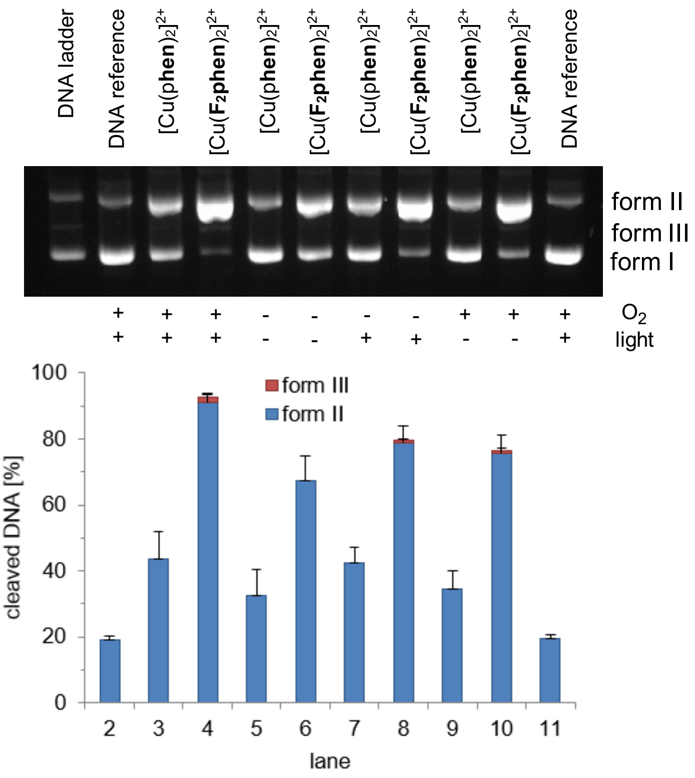

Figure S-5.4. Graphical representation of cleavage and representative gel of pBR322 plasmid DNA (0.025 µg µL^{-1}) with [Cu(**phen**)$_2$]$^{2+}$ and [Cu(**F$_2$phen**)$_2$]$^{2+}$ (100 µM) in 50 mM MOPS (pH 7.4) under normal and inert gas atmosphere as well as in the presence and absence of light after 60 min incubation at 37 °C. Lane 1: DNA ladder (form I, II and III), lane 2: DNA reference (t = 60 min), lane 11: DNA reference (t = 0 min).

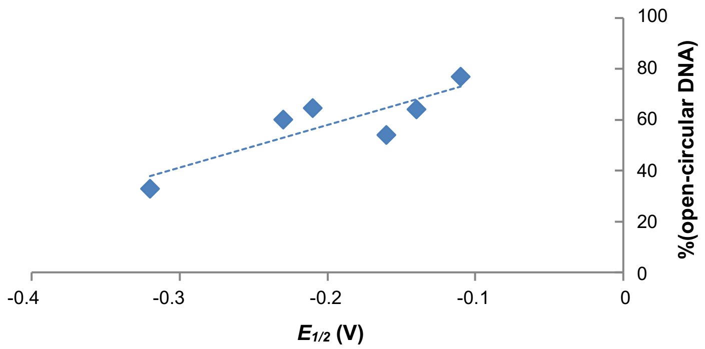

Figure S-5.5. Percentage of open-circular DNA (as determined from Figure 4) *vs.* half-wave potentials $E_{1/2}$ of Cu(II) complexes. Note: The electrochemical reaction for Cu(II)→Cu(I) transition relies on an outer-sphere electron transfer process at the heterogenous electrode surface, whereas the reaction between Cu(II) and a reducing agent in solution represents an inner-sphere process. Such an electron transfer does not necessarily have to proceed via an outer-sphere mechanism. Thus, even though there can be direct correlations between electrochemical and chemical redox reactions, it does not always have to be the case.[14]

Table S-5. Association constants for Cu(II) buffer adducts and the Cu(II) phenanthroline system.

Complex	Reaction	Association constant (log *K*)
[Cu(**phen**)]$^{2+}$	Cu^{2+} + **phen** ⇄ [Cu(**phen**)]$^{2+}$	8.8[15]
[Cu(**phen**)$_2$]$^{2+}$	[Cu(**phen**)]$^{2+}$ + **phen** ⇄ [Cu(**phen**)$_2$]$^{2+}$	6.5[15]
[Cu(Tris)]$^{2+}$	Cu^{2+} + Tris ⇄ [Cu(Tris)]$^{2+}$	5.34–6.29[16]
[Cu(MOPS)]$^{2+}$	Cu^{2+} + MOPS ⇄ [Cu(MOPS)]$^{2+}$	no coordination observed[17]

S-6 DNA melting curves

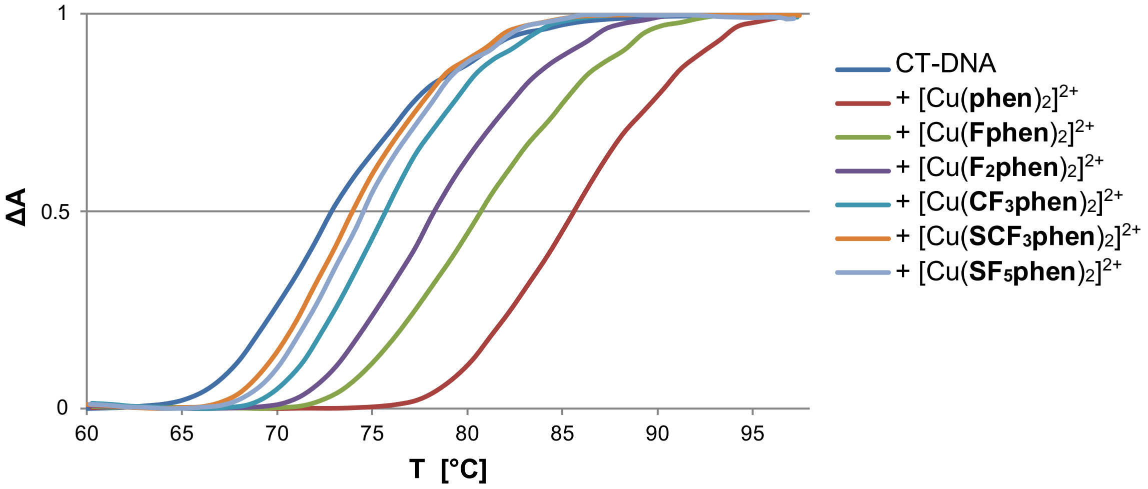

Figure S-6. DNA melting curves with CT-DNA (250 µM) and Cu(II) complexes (25 µM) in Tris-HCl (20 mM, pH = 7.4).

Table S-6. DNA melting temperatures as determined from the curves in Figure S-6.

	T_m	ΔT_m
CT-DNA	73.0 ± 0.1 °C	-
+ [Cu(**phen**)$_2$]$^{2+}$	85.7 ± 0.1 °C	12.7 ± 0.2 °C
+ [Cu(**Fphen**)$_2$]$^{2+}$	80.7 ± 0.1 °C	7.7 ± 0.2 °C
+ [Cu(**F$_2$phen**)$_2$]$^{2+}$	78.1 ± 0.2 °C	5.1 ± 0.3 °C
+ [Cu(**CF$_3$phen**)$_2$]$^{2+}$	75.7 ± 0.1 °C	2.7 ± 0.2 °C
+ [Cu(**SCF$_3$phen**)$_2$]$^{2+}$	74.3 ± 0.3 °C	1.3 ± 0.4 °C
+ [Cu(**SF$_5$phen**)$_2$]$^{2+}$	74.8 ± 0.2 °C	1.8 ± 0.3 °C

S-7 Ethidium bromide displacement

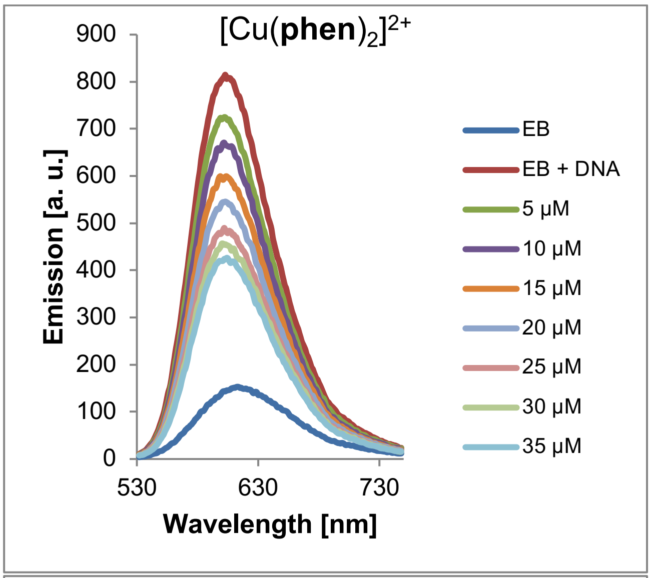

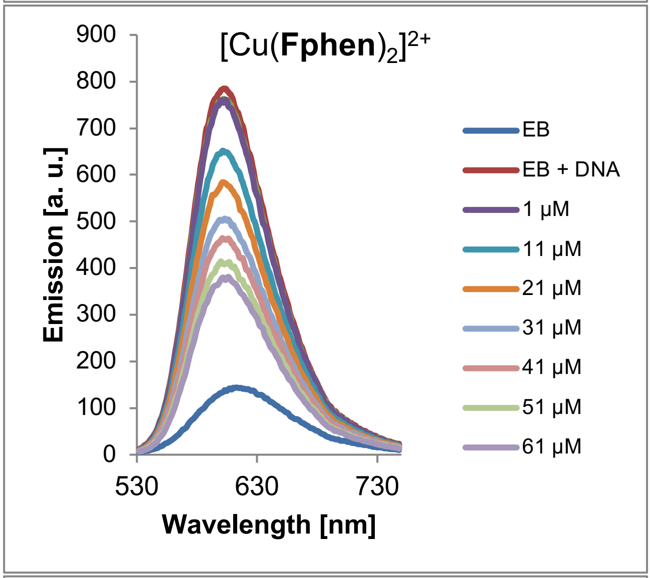

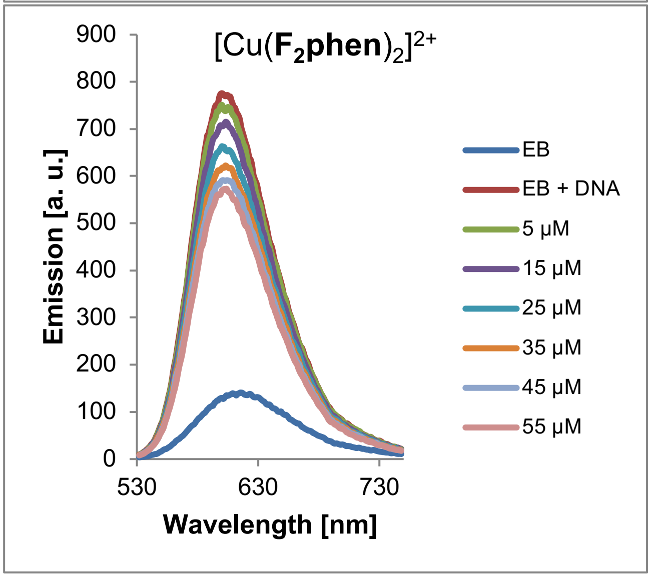

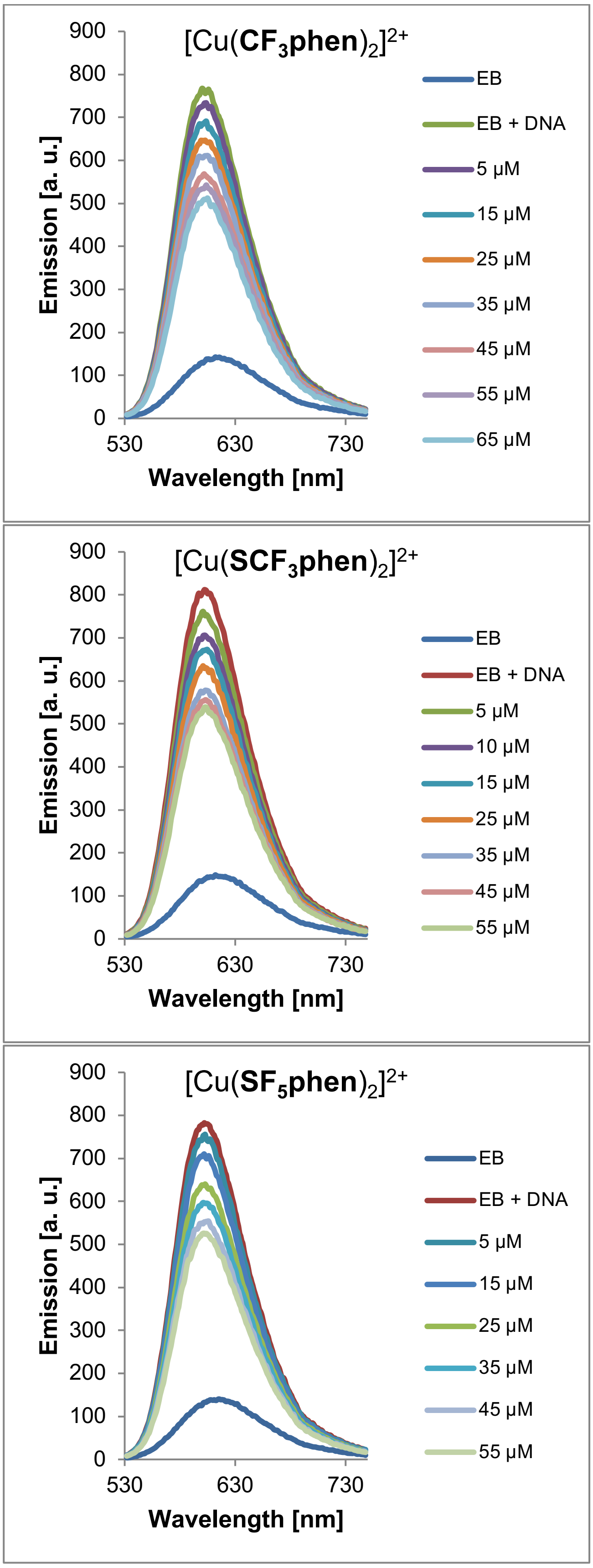

Figure S-7. EB displacement of the EB-CT-DNA system (20 μM CT-DNA; 5 μM EB) in Tris-HCl buffer (10 mM, pH 7.4) and titration with Cu(II) complexes $[Cu(\mathbf{Xphen})_2]^{2+}$ (X = H, F, 2xF, CF_3, SCF_3, SF_5).

S-8 MTT assay

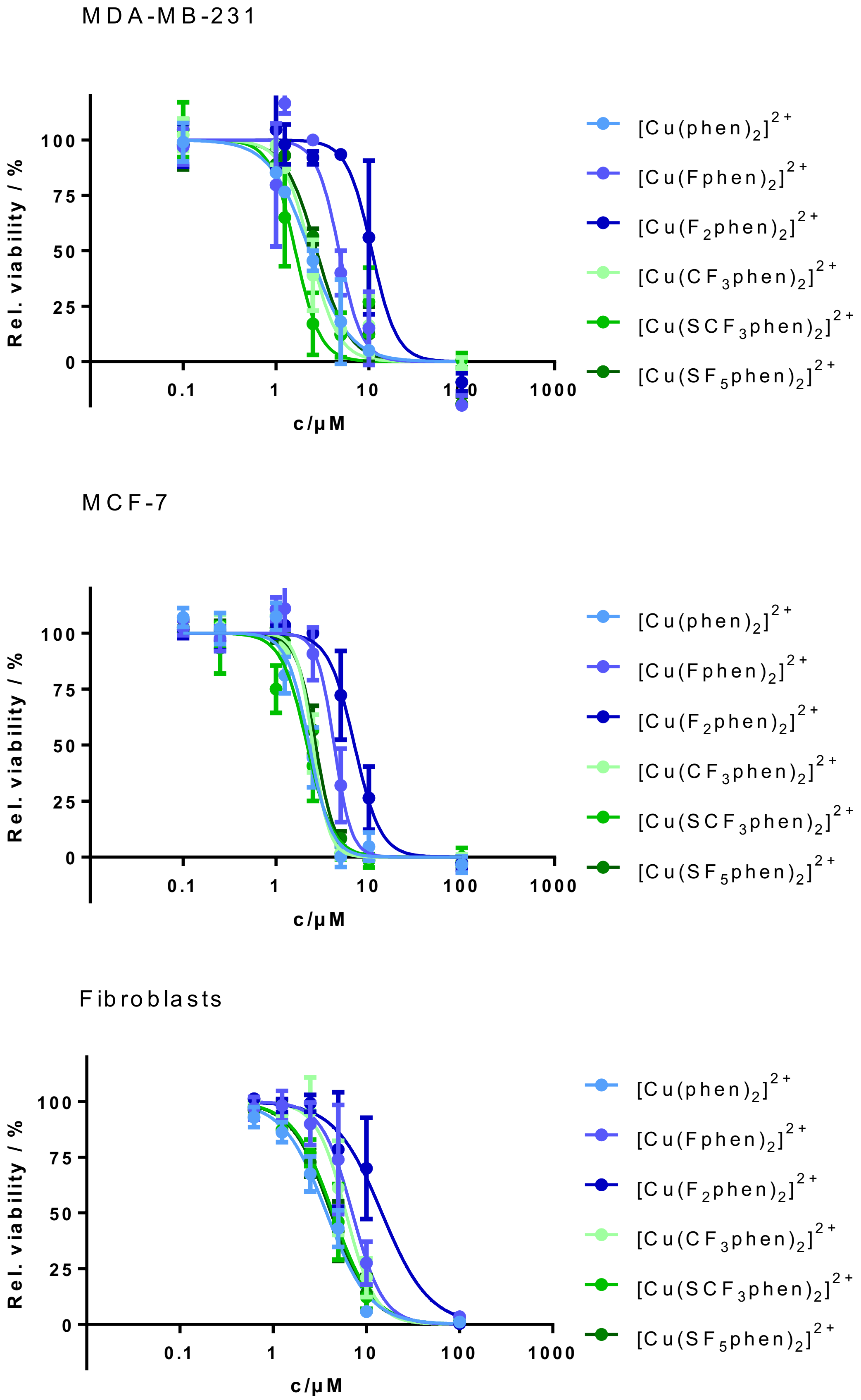

Figure S-8. Dose/viability curves of cells after 48 h incubation with increasing concentrations of compounds, determined by MTT assay.

Table S-8. Complete list of IC_{50} values and 95% confidence intervals (CI) obtained by the MTT assay.

**MDA-MB-231**	IC_{50} [µM]	95% CI
[Cu(**phen**)$_2$]$^{2+}$	2.3	1.7 to 3.1
[Cu(**Fphen**)$_2$]$^{2+}$	4.8	3.2 to 7.1
[Cu(**F$_2$phen**)$_2$]$^{2+}$	10.8	6.8 to 17.2
[Cu(**CF$_3$phen**)$_2$]$^{2+}$	2.3	1.8 to 3.1
[Cu(**SCF$_3$phen**)$_2$]$^{2+}$	1.6	1.2 to 2.2
[Cu(**SF$_5$phen**)$_2$]$^{2+}$	2.7	2.1 to 3.6

**MCF-7**	IC_{50} [µM]	95% CI
[Cu(**phen**)$_2$]$^{2+}$	2.3	2.0 to 2.7
[Cu(**Fphen**)$_2$]$^{2+}$	4.2	3.5 to 5.1
[Cu(**F$_2$phen**)$_2$]$^{2+}$	7.0	5.7 to 8.7
[Cu(**CF$_3$phen**)$_2$]$^{2+}$	2.5	2.3 to 2.7
[Cu(**SCF$_3$phen**)$_2$]$^{2+}$	2.2	1.8 to 2.6
[Cu(**SF$_5$phen**)$_2$]$^{2+}$	2.7	2.4 to 2.9

**Fibroblasts**	IC_{50} [µM]	95% CI
[Cu(**phen**)$_2$]$^{2+}$	3.7	3.2 to 4.4
[Cu(**Fphen**)$_2$]$^{2+}$	7.1	5.4 to 9.4
[Cu(**F$_2$phen**)$_2$]$^{2+}$	14.7	6.7 to 32.5
[Cu(**CF$_3$phen**)$_2$]$^{2+}$	6.0	4.7 to 7.8
[Cu(**SCF$_3$phen**)$_2$]$^{2+}$	4.4	3.6 to 5.4
[Cu(**SF$_5$phen**)$_2$]$^{2+}$	4.2	3.4 to 5.1

S-9 Determination of the partition coefficient log *P*

Table S-9. n-Octanol-water partition coefficients (log *P* values) of Cu(II) complexes.

compound	log *P*
[Cu(**phen**)$_2$]$^{2+}$	-1.72
[Cu(**Fphen**)$_2$]$^{2+}$	-1.04
[Cu(**CF$_3$phen**)$_2$]$^{2+}$	-0.27
[Cu(**SCF$_3$phen**)$_2$]$^{2+}$	-0.09
[Cu(**SF$_5$phen**)$_2$]$^{2+}$	-0.05
[Cu(**F$_2$phen**)$_2$]$^{2+}$	-0.36

S-10 Stability tests

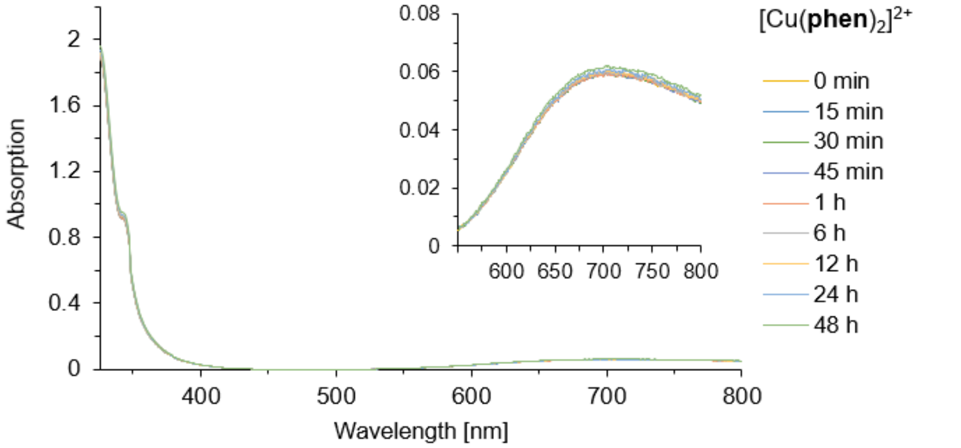

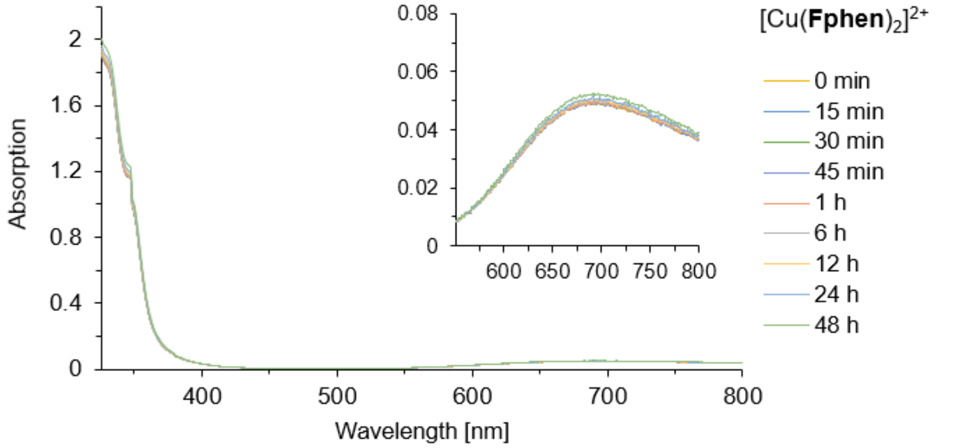

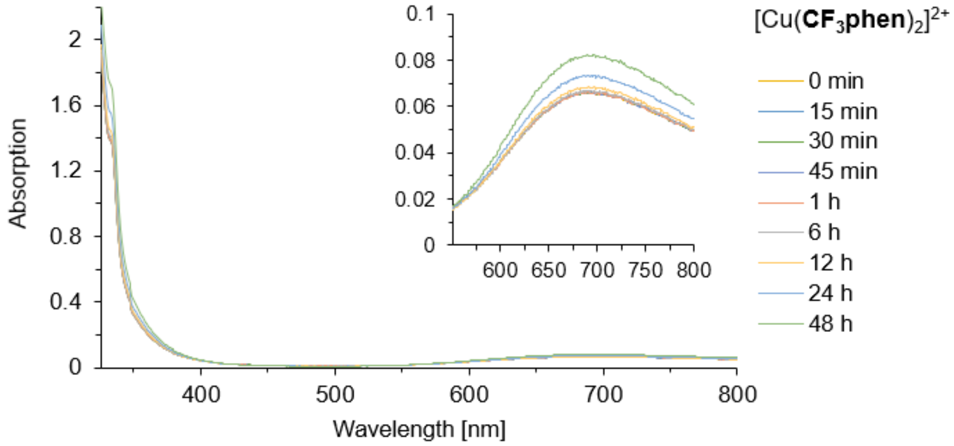

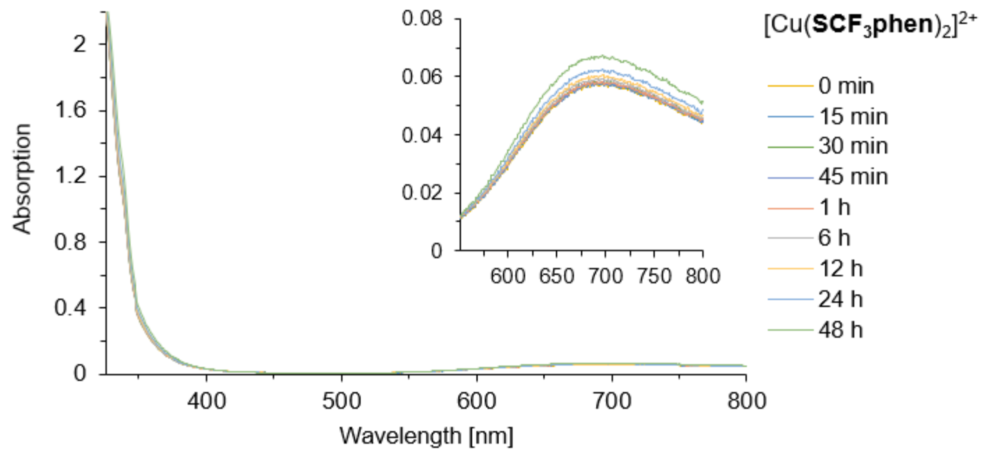

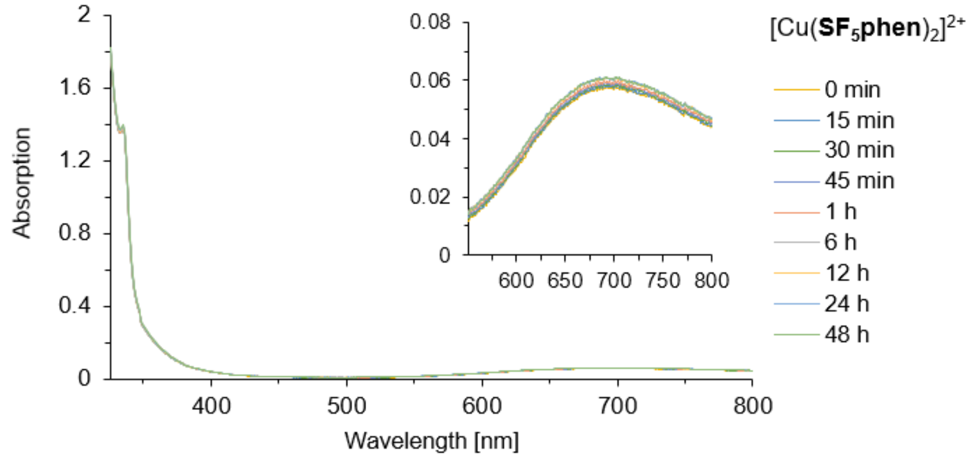

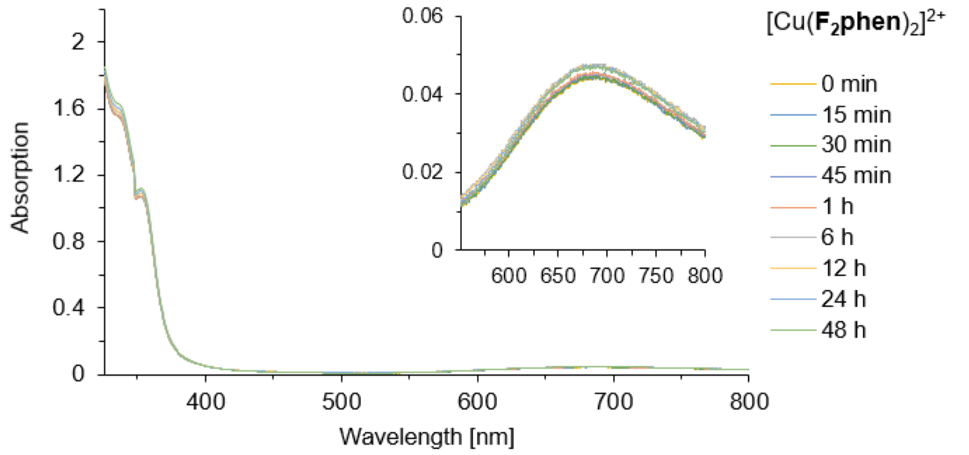

Figure S-10. Absorbance of Cu(II) complexes $[Cu(\mathbf{Xphen})_2]^{2+}$ (X = H, F, 2xF, CF_3, SCF_3, SF_5) (1 mM) at 37 °C and pH 7.4 (50 mM MOPS) monitored at different time points as indicated in the graphs.

References

[1] C. Lüdtke, A. Haupt, M. Wozniak, N. Kulak, *J. Fluorine Chem.* **2017**, *193*, 98–105.
[2] M. Boutebdja, A. Lehleh, A. Beghidja, Z. Setifi, H. Merazig, *Acta Cryst.* **2014**, *E70*, m185–m186.
[3] *SADABS*; Bruker AXS Inc.: Madison, WI, **2001**.
[4] G. M. Sheldrick, *Acta Crystallogr.* **2015**, *A71*, 3–8.
[5] G. M. Sheldrick, *Acta Crystallogr.* **2015**, *C71*, 3–8.
[6] O. V. Dolomanov, L. J. Bourhis, R. J. Gildea, J. A. K. Howard, H. Puschmann, *J. Appl. Crystallogr.* **2009**, *42*, 339–341.
[7] M. E. Reichmann, S. A. Rice, C. A. Thomas, P. Doty, *J. Am. Chem. Soc.* **1954**, *76*, 3047–3053.
[8] J.-A. Cuello-Garibo, M. S. Meijer, S. Bonnet, *Chem. Commun.* **2017**, *53*, 6768–6771.
[9] C. Al Hageh, M. Al Assaad, Z. El Masri, N. Samaan, M. El-Sibai, C. Khalil, R. S. Khnayzer, *Dalton Trans.* **2018**, *47*, 4959–4967.
[10] R. R. Gagné, C. A. Koval, G. C. Lisensky, *Inorg. Chem.* **1980**, *19*, 2854–2855.
[11] A. R. Morgan, J. S. Lee, D. E. Pulleyblank, N. L. Murray, D. H. Evans, *Nucleic Acids Res.* **1979**, *7*, 547–565.
[12] R. P. Hertzberg, P. B. Dervan, *Biochemistry* **1984**, *23*, 3934–3945.
[13] D. D. Stöbener, M. Uckert, J. L. Cuellar-Camacho, A. Hoppensack, M. Weinhart, *ACS Biomater. Sci. Eng.* **2017**, *3*, 2155–2165.
[14] J. Schnödt, M. Sieger, B. Sarkar, J. Fiedler, J. S. Manzur, C.-Y. Su, W. Kaim, *Z. Anorg. Allg. Chem.* **2011**, *637*, 930–934.
[15] B. R. James, R. J. P. Williams, *J. Chem. Soc.* **1961**, 2007–2019.
[16] J. Nagaj, K. Stokowa-Sołtys, E. Kurowska, T. Frączyk, M. Jeżowska-Bojczuk, W. Bal, *Inorg. Chem.* **2013**, *52*, 13927–13933.
[17] H. E. Mash, Y.-P. Chin, L. Sigg, R. Hari, H. Xue, *Anal. Chem.* **2003**, *75*, 671–677.
